# Gua-Sha therapy on breast engorgement among Indian postnatal mothers

**DOI:** 10.6026/97320630019853

**Published:** 2023-08-31

**Authors:** Amudha N, Prakash D

**Affiliations:** Department of Obstetrics and Gynecology Nursing, Indira College of Nursing, Trichy-621105, Tamil Nadu, India; Department of Medical Surgical Nursing, Nootan College of Nursing, Sankalchand Patel University, Visnagar, Gujarat-384315, India

**Keywords:** Gua-Sha therapy, breast engorgement, pain, postnatal mothers

## Abstract

Many women experience breast engorgement in the first few weeks after giving birth. Breast engorgement that is somewhat severe. It is characterized by full, tense, heated, and tender breasts that are painfully throbbing and aching. Therefore, it is of interest to evaluate the effect of Gua-Sha therapy on breast engorgement in reducing pain among postnatal mothers. A "non-randomized pre-test post-test control group design" was used for this study. "Purposive sampling techniques" were used to obtain 60 postnatal mothers who satisfied the inclusion criteria. Six point engorgement scale and visual analog scale were used for data collection. After pre-test Gua-Sha therapy was given 30 minutes in one cycle twice a day depending on upon the severity. Reassessment was done immediately after the procedure. The result shows that the post test score of breast engorgement in experimental group was 1.1 (± 0.305), where in control group 4.16 (±2.152). The 't' test value of breast engorgement was 9.869. The result shows that Gua-Sha therapy for breast engorgement in reducing pain was significant effect (p <0.05). The study concluded that the Gua-Sha therapy is an effective for reducing breast engorgement and pain among post natal mothers.

## Background:

There is no alternative for mother's milk; breastfeeding is a mother's gift to her child, to the environment, and to herself. A baby needs antibodies and essential nutrients immediately after delivery, which is found in the yellowish milk known as colostrum [1]. Small amounts of colostrum are produced by mother breasts during the early stages of nursing. However, they will start to produce more milk after a few days. Consequently, breasts get bigger and firmer. In addition to more milk being produced, higher blood flow and additional lymph fluids in breast tissue also contribute to this swelling [2, 3]. When their baby is eating properly and frequently, these sensations of heaviness usually disappear for most new mothers without any issues. Engorgement, a condition when a breast produces more milk than it can hold, causes some women to feel uncomfortable and rock hard full. Breast feeding women frequently experience breast engorgement, which can cause painful blebs, clogged milk ducts, or mastitis [4], among other potentially serious problems. Breast engorgement affects a large number of nursing women [5]. A new born may find it challenging to latch on and feed correctly if they are severely engorged. Engorgement, often known as milk fever, may even result in a body temperature increase of 99-100 degrees Fahrenheit. Breast engorgement is described as the swell and distension of the breasts by the Academy of Breastfeeding Medicine Protocol Committee [6]. Usually in the first few days after lactation begins, brought on by vascular dilatation and the advent of early milk. Breast engorgement can also happen as a result of delayed, infrequent, or interrupted milk evacuation from the breast during the first week of breastfeeding [7, 8]. Failure to prevent or treat milk stasis brought on by irregular or insufficient breast drainage is one of the reasons that could increase a mother's risk of engorgement [9, 10]. Gua-Sha is an alternative therapy that involves using a massage tool to scrape your skin in order to increase circulation. This traditional Chinese therapeutic method might be a novel way to achieve improved health. In Gua-Sha, the skin is scraped in either short or lengthy strokes to promote the microcirculation of the soft tissue, which increases blood flow. They use a smooth-edged tool called as a Gua-Sha massage tool to apply these strokes. Gua-Sha is meant to treat the body's chi, or stagnated energy, which practitioners say may be the cause of inflammation. Numerous disorders connected to persistent pain are rooted in inflammation. It is believed that rubbing the skin's surface might assist break up this energy, lessen inflammation, and accelerate healing.

##  Methodology:

**Figure 1:**
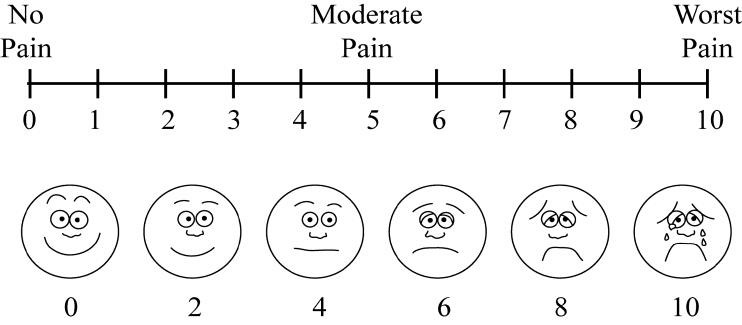
visual analogue scale

The study's research methodology, a quasi-experimental non-randomized control group design, was chosen. Using a non-probability purposive sampling technique, 60 postnatal women who satisfied the inclusion criteria were gathered. The institutional authority of the maternity hospital gave formal consent before starting the investigation. In order to obtain their written agreement to be enrolled in the study as well as their assistance, the researcher introduced herself to postnatal mothers and explained the purpose of the study. Prenatal women provided information on obstetrical variables such parity, birth method, postnatal day, type of new born, type of nipple, start of breastfeeding, frequency of breastfeeding, feeding position, breastfeeding pattern, and mode of breastfeeding. The degree of breast engorgement is assessed using the six-point engorgement scale developed by Hill PD and Hume Nick (Table 1). Polit and Beck developed the standard scale known as the "visual analogue scale" to measure the intensity of pain. There were 6 categories, and there were a total of 10 potential scores. The calculation and interpretation of each subject score were as follows (Figure 1).

Three stages of data collecting were used: assessment, implementation and evaluation phase.

## Assessment phase:

[1] Data were gathered using an interview schedule, which was completed individually and in complete secrecy.

[2] On the fourth postpartum day, study subjects with verified breast engorgement were interviewed in the study settings to collect data.

[3] The researcher chose the mild and short Gua-Sha friction protocol out of three various types of Gua-Sha friction procedures (hard, moderate, and soft).

**Figure 2:**
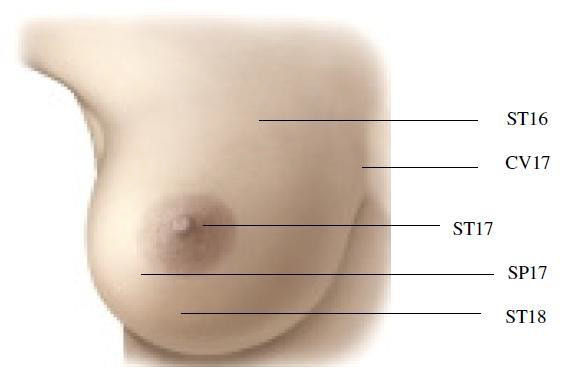
Gua-Sha technique for breast engorgement

[4] In terms of the intervention group, the researcher taught the intervention group how to apply the Gua-Sha technique, which contained acupoint positions ST16, ST18, SP17, and CV17 (Figure 2).

**Figure 3:**
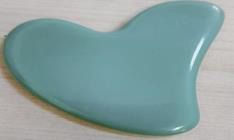
Gua-Sha equipment

**Figure 4:**
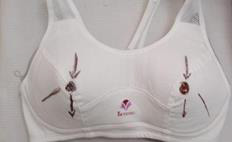
Gua-Sha supportive bra

[5] The researcher provided each woman with equipment to help them perform the technique, which included a Gua-Sha device (Figure 3) and a supportive bra based on breast size (small-medium-large) that was drawn in a direction to facilitate technique application (Figure 4).

## Implementation phase:

Experimental group:

The following tasks were assigned to women:

[1] Step 1: Wash hands

[2] Step 2: Get into bed.

[3] Step 3: Identify the engorged breast.

[4] Step 4: Put on the researcher's provided supportive bra.

[5] Step 5: Select the scraping points according to the direction of the supportive bra ST16, ST17, ST18, SP17, CV17.

[6] Step 6: Scrape each site three times for two minutes each, for 30 minutes per cycle.

[7] Step 7: Repeat the procedure twice daily till the breast softens.

## Control group:

The women in the control group were told to use normal care such as compresses and combing their breasts.

## Evaluation phase:

The degree of breast engorgement within each group was evaluated before and after intervention, and differences between the two study groups were established. "Statistical Package for the Social Sciences (SPSS) version 22" was applied to enter the data, analyze it, and draw conclusions. For categorical variables, frequency and percentages were determined in addition to mean and standard deviation for numerical variables. "T test" for test hypothesis and chi square for find association between post-test score with demographic variables.

## Results:

Table 2 shows that demographic variables majority of the subjects 14 (46.7%) were in the age group of 18-25 years in experimental group and 13 (43.3%) were in the age group of 26-32 years in control group, based on education 16 (53.3) were graduate and above in experimental group and 21 (70%) were graduate and above in control group. According to occupation 17 (56.7%) were working in experimental group and 17 (56.7%) were working women in control group. Based on type of family 20 (66.7%) were nuclear family in experimental group and 18 (60%) were nuclear family in control group, according to food habits 17 (56.7%) were vegetarian in experimental group and 18 (60%) were vegetarian in control group. According to the obstetrical variables majority of the subjects 14 (46.7%) were parity of two in experimental group and 16 (53.3%) were in the parity of two in control group, based on mode of delivery 16 (53.3%) were caesarean section in experimental group and 15 (50%) were caesarean section in control group. In initiation of breast feeding 16 (53.3%) were >1 hour in experimental group and 17(56.7%) were >1 hour in control group, according to post natal days 21 (70%) were 1-5 days in experimental group and 24 (80%) were 1-5 days in control group. According to type of new born 25 (83.3%) were term in experimental group and 27 (90%) were term in control group. Based on type of nipple 13 (43.3%) were flat nipple in experimental group and 12 (40%) were normal nipple in control group. In frequency of feeding 19(63.3%) were demand feeding in experimental group and 17 (56.7%) were demand feeding in control group. According to breast feeding pattern at each time 18 (60%) were one side breast in experimental group and 19 (63.3%) were one side breast in control group. In mode of breast feeding 26 (86.7%) were direct breast feeding in experimental group and 28 (93.3%) were direct breast feeding in control group.

**Figure 5:**
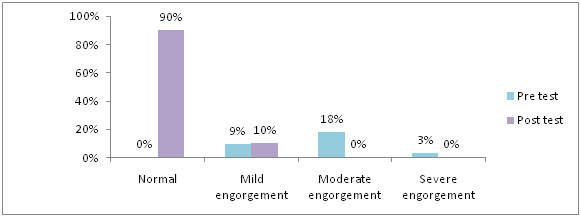
Distribution of samples based on level of breast engorgement in experimental group

**Figure 6:**
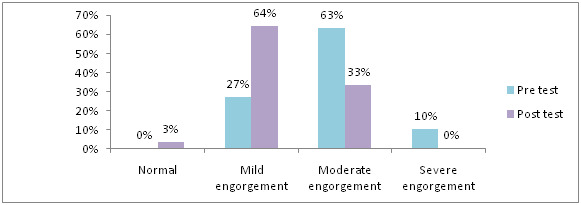
Distribution of samples based on level of breast engorgement in control group

**Figure 7:**
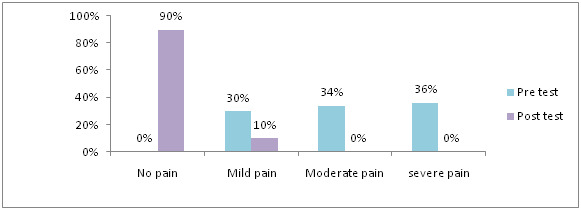
Distribution of samples based on level of breast pain in experimental group

**Figure 8:**
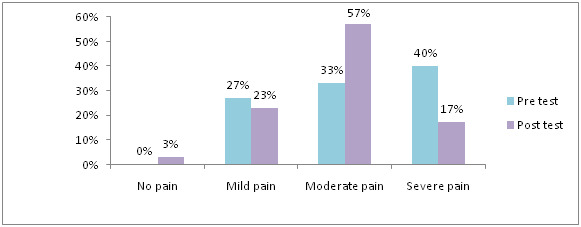
Distribution of samples based on level of breast pain in control group

Figure 5 shows that, in pre-test 3% had severe engorgement, 18% had moderate engorgement, 9% had mild engorgement and in post-test 90% never feel any pain, 10% mild pain, 18% had moderate engorgement, in experimental group. Figure 6 shows that, in pre-test 10% had severe engorgement, 63% had moderate engorgement, 64% had mild engorgement and in post-test 3% never feel any pain, 64% had mild pain, 33% had moderate engorgement, in control group. Figure 7 shows that, in pre-test 30% had mild pain, 34% had moderate pain, 36% had severe pain and in post-test 90 % never feel any pain, 10 % had mild pain in experimental group. Figure 8 shows that majority 90% had no pain, 27 % had mild pain, 33% had moderate pain and 40% had severe pain seen in pre-test. After application of Gua-Sha therapy 17% had severe pain 57% had moderate pain, 23% had mild pain, 3% had no pain in control group.

Table 3 shows mean post test score of level of breast engorgement among experimental group was 1.1 (± 0.480), where as in control group it was 3.066 (± 0.980). The mean post test score of pain was 0.1 (± 0.305), where as in control group it was 4.16 (± 2.152). The 't' value of level of breast engorgement was 9.869 and for pain were 10.231 respectively. It shows the Gua-Sha therapy has significant effect (P < 0.05) in reducing breast engorgement and pain.

## Discussion:

The aim of the study was to evaluate the effectiveness of Gua-Sha therapy on breast engorgement in reducing pain and to find out the association between the post-test levels of breast engorgement with selected demographic variables among post natal mothers. A study conducted by Chiu JY in Taiwan and the findings showed that Gua-Sha therapy may be used as an effective technique in the management of breast engorgement. By using Gua-Sha therapy, nurses can handle breast engorgement problems more effectively in primary care and hence help patients both physically and psychologically [11]. Another study conducted by Neethu V and her study findings indicated that there is a significant relief in breast engorgement after the implementation of Gua-Sha therapy. Hence, she concluded that Gua-Sha therapy is effective in relieving breast engorgement among post natal mothers [12]. Mary selected 54 women who had uncomplicated births and were diagnosed with engorgement. The intervention group received 7 light unidirectional strokes starting at 1 of 3 meridians from the base of the breast toward the nipple for 2 cycles, which took about 2 minutes. She concluded that Gua-Sha therapy was more effective in reducing pain and breast engorgement [13]. Fatemeh concluded that the Gua-Sha therapy, oketani massage, and hollyhock leaf compress decreased the mean severity of breast engorgement [14]. Mangesi showed that some interventions, such as hot/cold packs, Gua-Sha, acupuncture, cabbage leaves, and proteolytic enzymes, appear promising for treating breast engorgement during lactation, there is insufficient evidence from published trials to justify widespread implementation [15]. W Kamal Ali Farag findings revealed that women who practiced the Gua-Sha technique during the early postpartum period exhibit less breast engorgement than women received routine care. She explained that he Gua-Sha technique should be recommended as a safe non-pharmacological method in treating breast engorgement and include it as part of postpartum women's discharge teaching plan [16]. Saumya discussed that the prevalence of breast engorgement is common now a days and appropriate treatment should be given at the earliest as this may interfere the breast feeding and thus causes mal nourishment and other issues during the early developmental period. Cold compression, massage therapy and Gua-Sha therapy are safe and can be advised to treat the symptoms of breast engorgement [17].

## Conclusion:

The incidence of breast engorgement is now widespread, and prompt treatment should be offered as it may interfere with breastfeeding and lead to malnutrition and other problems during the early stages of development. The results of the study led to the conclusion that Gua-Sha therapy is useful for lowering breast engorgement and pain in postpartum women and those moms can also try other complementary therapies including cabbage leaves, breast massage, and garlic.

## Figures and Tables

**Table 1:** Six-point breast engorgement scale

**Score**	**Description**
1	Soft and no changes in breasts
2	Slight changes in breasts
3	Firm and non-tenderness breasts
4	Firm and beginning tenderness in breasts
5	Firm and tender
6	Very firm and very tender

**Table 2:** Demographic and obstetric variables among post-natal mothers

**No**	**Variables**	**Experimental group**		**Control group**	
		**Frequency**	**Percentage**	**Frequency**	**Percentage**
1	**Age:**
	18-25	14	46.7	10	33.3
	26-32	10	33.3	13	43.3
	>32	6	20	7	23.3
2	**Education:**
	Illiterate	0	0	0	0
	Primary	5	16.7	2	6.7
	Secondary	9	30	7	23.3
	Graduate & above	16	53.3	21	70
3	**Occupation:**
	House Wife	13	43.3	13	43.3
	Working	17	56.7	17	56.7
4	**Types of Family:**				
	Nuclear	20	66.7	18	60
	Joint	10	33.3	12	40
5	**Food Habits:**
	Vegetarian	17	56.7	18	60
	Non Vegetarian	13	43.3	12	40
6	**Parity:**
	One	12	40	8	26.7
	Two	14	46.7	16	53.3
	More than Two	4	13.3	6	20
7	**Mode of Delivery:**
	Vaginal Delivery	13	43.3	13	43.3
	Forceps Delivery	1	3.3	2	6.7
	Caesarean Delivery	16	53.3	15	50
8	**Initiation of Breast Feeding:**
	Within 1 hour	14	46.7	13	43.3
	>1 hour	16	53.3	17	56.7
	None of the above	0	0	0	0
9	**Post Natal period:**
	5-Jan	21	70	24	80
	10-Jun	6	20	4	13.3
	>10	3	10	2	6.7
10	**Type of New Born:**
	Term	25	83.3	27	90
	Pre-Term	4	13.3	1	3.3
	Post-term	1	3.3	2	6.7
	Still Birth	0	0	0	0
11	**Type of Nipple:**
	Normal Nipple	7	23.3	12	40
	Flat Nipple	13	43.3	9	30
	Inverted Nipple	10	33.3	9	30
12	**Frequency of Feeding:**
	Every 2 hours	11	36.7	13	43.7
	Demand feeding	19	63.3	17	56.7
	None of the above	0	0	0	0
13	**Breast Feeding Pattern at each time:**
	One side breast	18	60	19	63.3
	Both breast	12	40	11	36.7
	None of the above	0	0	0	0
14	**Mode of Breast Feeding:**
	Expressed breast feeding	4	13.3	2	6.7
	Direct breast feeding	26	86.7	28	93.3
	None of the above	0	0	0	0

**Table 3:** Effectiveness of Gua-Sha therapy on breast engorgement in reducing pain among postnatal mothers

**S. No**	**Variables**	**Experimental group**		**Control group**		**T Test**
		Mean	SD	Mean	SD	
1	Breast engorgement	1.1	0.48	3.066	0.98	9.869
2	Pain	0.1	0.305	4.36	2.152	10.231
